# Relevance of the Consensus Principles for Appropriate Antibiotic Prescribing in 2022

**DOI:** 10.1093/jac/dkac211

**Published:** 2022-09-06

**Authors:** Rafael Cantón, Murat Akova, Karen Langfeld, Didem Torumkuney

**Affiliations:** Hospital Universitario Ramón y Cajal and Instituto Ramón y Cajal de Investigación Sanitaria (IRYCIS) Madrid, Madrid, Spain; CIBER de Enfermedades Infecciosas, Instituto de Salud Carlos III, Madrid, Spain; Faculty of Medicine, Department of Infectious Diseases and Clinical Microbiology, Hacettepe University, Sihhiye, Ankara, Türkiye; GlaxoSmithKline, 980 Great West Road, Brentford, Middlesex TW8 9GS, UK; GlaxoSmithKline, 980 Great West Road, Brentford, Middlesex TW8 9GS, UK

## Abstract

**Background:**

In the late 1990s, as a response to rising antimicrobial resistance (AMR), an independent multinational, interdisciplinary group was formed specifically targeting primary care antibiotic prescribing for community-acquired respiratory tract infections (CA-RTIs). The group comprised senior clinicians from Canada, Israel, Spain, Sweden, UK and USA. The group’s objectives were to provide recommendations for antibiotic stewardship in the community because, whilst it was widely accepted that inappropriate antibiotic use was contributing to AMR, it remained difficult to change prescribing behaviour. The group aimed to identify principles underlying appropriate antibiotic prescribing and guideline formulation to reduce morbidity from CA-RTIs, limit therapeutic failure and, importantly, curb AMR emergence. The group published a report in 2002, which has become known as the Consensus Principles.

**Objectives:**

(i) To consider the relevance of the Consensus Principles in 2022 by reviewing current global approaches to rising AMR. A wide range of factors, such as antibiotic overuse, most recently seen in COVID-19 patients, are still driving rising AMR even though there has been a high-level international response to the AMR threat; and (ii) as an introduction to this Supplement, which reports the findings of analyses of how AMR is being addressed in nine disparate countries (Brazil, India, Kuwait, Mexico, Pakistan, Russia, Saudi Arabia, Türkiye and Vietnam). Understanding how these initiatives are being pursued in different countries helps identify areas where more information is needed.

**Conclusions:**

Adherence to the Consensus Principles remains as important now as it was in 2002. Achieving appropriate antibiotic prescribing is a vital objective in order that the right patient receives the right antibiotics at the right time to ensure optimal clinical outcomes while at the same time helping to limit further increases in AMR.

## Introduction

The World Health Organization (WHO) describes antimicrobial resistance (AMR) as one of the biggest threats to global public health,^1^ stating that ‘the world urgently needs to change the way it prescribes and uses antibiotics. Even if new medicines are developed, without behaviour change, antibiotic resistance will remain a major threat’.^2^ New antibiotic resistance mechanisms threaten our ability to treat common bacterial infections and, without urgent action, we are heading for a post-antibiotic era, in which common infections and minor injuries could once again be fatal.^1,2^

Without effective antimicrobials, management of infections in critically ill patients (and those with various immunosuppressed conditions including, but not restricted to, organ transplantation, cancer chemotherapy, uncontrolled diabetes or major surgery) would be extremely difficult and sometimes even impossible.^1,2^ It has been widely demonstrated that AMR increases healthcare costs due to prolonged hospital stays, increased intensive care requirements and the need for additional antibiotics.^1,2^

Although the development of AMR is a natural phenomenon, the selection of resistant bacterial strains is driven by antibiotic use in both humans and animals.^1–5^ Other circumstances leading to the emergence and spread of AMR include global travel, poor sanitation, shortage of rapid diagnostic facilities, excessive antibiotic use in animal husbandry, environmental spillages, overprescribing of broad-spectrum antibiotics, antibiotic misuse, inappropriate antibiotic prescribing and use of poor quality antibiotics.^3–5^

The ongoing global COVID-19 pandemic has added an additional and very concerning dimension to this scenario. The incidence of bacterial co-infection or superinfection in COVID-19 patients (who are suffering from a viral infection) is low and the prevalence of antibiotic prescribing amongst these patients is high, as shown by a large meta-analysis in which the estimated bacterial co-infection rate in COVID-19 patients was 8.6% but the prevalence of antibiotic prescribing was 74.6%.^6^ In addition, in the early part of the pandemic, azithromycin was prescribed for a high proportion of COVID-19 patients as it was considered to be a potentially therapeutic agent. Concerns have been raised that this over-prescribing of azithromycin during the COVID-19 pandemic could encourage an increase in resistance to macrolides.^7^ The elevated level of antibiotic prescribing in general during the pandemic has led many authors to warn of the possible concomitant risk of a rise in AMR.^8–13^

A further dynamic relates to the unusual position occupied by infectious diseases and their treatment. Unlike other medical conditions that involve the use of drugs that interact with biochemical pathways or physiological systems in the patient’s cells and tissues, antibiotics act on unique molecular targets present in the bacterial pathogen. Thus, antibiotic treatment involves ‘selective toxicity’ with the aim of eradicating the pathogen, or at least reducing the bacterial load, without harming the patient. Antibiotics are ‘societal’ drugs, as their use by an individual can affect other members of society. Unlike any other class of drug, use of antibiotics can result in their own reduced efficacy due to increasing resistance. This, in turn, can affect other members of society because the efficacy of the antibiotic has been diminished.^14–16^

## Discovery of antibiotics and antimicrobial resistance development

The modern age of antimicrobial therapy commenced in 1910 when the German physician and scientist Paul Ehrlich introduced Salvarsan as the first antimicrobial agent for the treatment of syphilis.^17^ The implications of AMR were, however, soon recognized and in 1945 in his Nobel Lecture, Sir Alexander Fleming cautioned not only on the perils of over-the-counter availability of antibacterial agents but also raised concerns about the possible future abuse of antibiotics. He warned how both overuse, and particularly underuse of penicillin would lead to resistance development and predicted what this would mean in terms of future treatment.^18^ Recent research reveals that genes for resistance to antimicrobial agents were present in bacteria at least three million years before evidence of humans; it is the recent use of antibiotics that has selected for these genes and led to the widespread development and transmission of resistance.^19,20^ During the period from the 1940s to the 1960s, all major antibiotic classes in use today were developed, including the aminoglycosides, β-lactams, macrolides, sulphonamides, glycopeptides, fluoroquinolones, tetracyclines and others.^21^ By the end of the 1980s, however, many pharmaceutical companies had reduced their antibiotic research investment resulting in a dramatic decrease in the number of new antibiotics being developed. This ‘Innovation Gap’ meant that no new classes of antibiotics were available to replace those rendered less useful due to resistance.^22,23^ Antibiotic research has continued to decline; whilst 18 large pharmaceutical companies had been actively involved in the area in 1990, by 2013 this number had dropped to just four.^23,24^

By the end of the twentieth century, it was acknowledged that increasing AMR amongst the main respiratory pathogens was compromising the therapy of the major respiratory syndromes, such as bacterial pneumonia and acute exacerbations of chronic obstructive pulmonary disease.^25^

## Response to AMR

Whilst it was generally accepted that inappropriate use of antibiotics had contributed to the emergence of resistant pathogens, it remained difficult to change prescribing behaviour. This reflected in part the fact that the profusion of guidelines for antibiotic prescribing attempting to address these issues offered varying recommendations. In an effort to address this issue, towards the end of the 1990s an independent multinational, interdisciplinary panel, the Consensus Group on Resistance and Prescribing in Respiratory Tract Infections, was formed to provide recommendations to help manage this situation.^25^ This international group comprised senior clinicians from Canada, Israel, Spain, Sweden, UK and USA who were united by their interest in infectious disease management and additionally by their concerns about how rising AMR could, if unchecked, seriously compromise clinical outcomes and the longevity of antibiotics.^25^

The objectives of the Consensus Group were to optimize antibiotic therapy in order to reduce morbidity, therapeutic failure and consequent added cost and prevent resistance emergence. Primary care antibiotic prescribing, in particular, was targeted as it accounted for the majority of antibiotic prescriptions, a high percentage of which were for respiratory tract infection (RTI).^25^ Primary care physicians were under pressure from many directions, such as peer groups, pharmaceutical marketing, regulatory bodies, formulary and guideline committees, but would welcome some general, over-arching prescribing principles rather than additional management guidelines. The aim was to achieve a selective reduction in inappropriate prescribing. The Consensus Group wanted to provide a way of preventing further increases in AMR by improving the quality of prescribing, targeting RTI and primary or outpatient clinic care and by informing and influencing governments, healthcare providers, physicians, patients and the media.^26^

The outcomes from the Consensus Group on Resistance and Prescribing in Respiratory Tract Infections were published in the *Journal of Antimicrobial Chemotherapy* in 2002 and included six principles for appropriate antibiotic prescribing, known as the ‘Consensus Principles’^25^ summarized as:

1. **Treat only bacterial infections.** High levels of prescribing for non-bacterial infections, e.g. the common cold, particularly in primary care, were noted and pressure from patients and parents along with constraints on physician time were suggested as the main reason.
2. **Optimize diagnosis and severity assessment.** Lack of availability of cost-effective diagnostic tests was identified as the reason for the persistence of the so-called ‘grey areas’ of confusing aetiology. Practical criteria were needed to identify bacterial infections requiring antibiotic therapy.
3. **Maximize bacterial eradication.** Antibiotic therapy should reduce maximally or eradicate the bacterial load since evidence confirmed that bacterial eradication should be the goal of antibiotic therapy.
4. **Recognize (and act on) local resistance prevalence.** Increasing high-level resistance in RTI pathogens must result in increased therapeutic failures but the extent of failure in community-acquired respiratory tract infections (CA-RTIs) in relation to resistance prevalence was unknown. Prescribing should be appropriate in the type and context of local resistance prevalence.
5. **Utilize pharmacodynamics to assist in the choice of effective agents and dosing.** Prescribing should be based on pharmacokinetic/pharmacodynamic (PK/PD) principles that can predict efficacy (bacterial eradication) and thus limit the emergence of resistance.
6. **Integrate local resistance, efficacy and cost-effectiveness.** Pharmacoeconomic analyses had confirmed that bacteriologically more-effective antibiotics can reduce overall management costs particularly concerning consequential morbidity and hospital admission.

## Ongoing response to AMR

### Bacterial disease prevalence

In order to gauge the relevance of the Consensus Principles today, some two decades later, we must consider progress in combating AMR during that time. In terms of disease prevalence, lower RTIs (LRTIs) are still a key area of focus; they remain, according to the most recent WHO global health estimates, the most common infectious cause of death worldwide. When all causes of death are considered, LRTIs are the fourth-most common, only currently exceeded by ischaemic heart disease, stroke and chronic obstructive pulmonary disease.^27^ CA-RTIs, in particular community-acquired pneumonia (CAP), are associated with considerable morbidity and/or mortality^28^ and are the most common reason for antibiotic prescriptions in adults,^29^ a pattern that is mirrored in paediatric medicine. In post-neonatal paediatrics (1 to 59 months), pneumonia is the most common cause of death; the WHO estimates that pneumonia is the cause of 12% of deaths in children aged from 1 month to 5 years.^30^ Acute otitis media (AOM) is one of the most common childhood diseases, with more than 80% of children being affected by the age of 5 years.^31^ AOM represents the most common indication for both antibiotic prescription and outpatient visits in children.^32^ High antibiotic prescription rates globally, in both adults and children, mean that the management of RTIs still plays a significant role in AMR development.

AMR itself has become an important cause of death. A recent investigation—the Global Research on Antimicrobial Resistance (GRAM) study—provides the most comprehensive global estimates of the burden of AMR to date and addresses in detail the state of play in 2019.^33^ That study estimated deaths attributable to AMR and deaths associated with AMR in 2019 for 204 countries and concluded that 4.95 million deaths were associated with bacterial AMR, of which 1.27 million deaths were directly attributable to bacterial AMR.^33^ The study estimated that the death rate attributable to resistance was highest in western sub-Saharan Africa and lowest in Australasia. LRTIs were responsible for 1.5 million deaths associated with resistance in 2019. If a comparison is made with all underlying causes of death in the Global Burden of Disease (GBD) 2019, AMR would have been the third-leading GBD Level 3 cause of death in 2019, only ischaemic heart disease and stroke accounted for more deaths that year.^33^

### Antibiotic susceptibility testing

The Consensus Principles stated the need for *local* antibiotic susceptibility testing (AST) data for the most common respiratory pathogens, which could then be used by prescribers to aid antibiotic choice when empirical treatment is needed and could also be used to inform local guideline development.^25^ At the time of publication of the Consensus Principles, some large surveillance studies on AMR were underway although global cover was very variable.^34^ Examples of early large-scale studies involving several centres and a range of antibiotics include the Alexander Project, the PROTEKT study and the SENTRY Antimicrobial Surveillance Program.^34^

#### Alexander Project

The Alexander Project, an international multicentre surveillance study of antimicrobial susceptibility of common respiratory pathogens, was initiated in 1992. The study was important because the data could be compared between different locations or centres or over time, to identify trends because a central testing laboratory and standard methods were used. The Alexander Project has been regarded as ‘pivotal’ for global surveillance studies.^35^

#### PROTEKT

PROTEKT (Prospective Resistant Organism Tracking and Epidemiology for Ketolide Telithromycin) which started in 1992, is also an international study which investigated the susceptibility of CA-RTI pathogens with, in a similar way to other studies, the focus being on *Streptococcus pneumoniae*, *Haemophilus influenzae* and *Moraxella catarrhalis.*^36^

#### SENTRY

The SENTRY Antimicrobial Surveillance Program (SENTRY) was started in 1997. The aim of the programme was to keep track, globally, of pathogens and resistance trends for both infections within hospitals and in the community and this was done through a network of hospitals. Pathogens from a range of infections are included e.g. bloodstream CA-RTI, skin and soft tissue infections (SSTI), gastroenteritis and others.^37^

#### ATLAS

The Antimicrobial Testing Leadership and Surveillance (ATLAS) programme is a global database of AMR data which can be searched using both isolate and patient criteria. ATLAS includes data covering the *in vitro* activity of antimicrobials against isolates from a range of infections such as RTIs, SSTIs, intestinal, musculoskeletal infections and others.^38^

#### SOAR

The Survey of Antibiotic Resistance (SOAR)^39^ is a long-running and extensive surveillance study which started in 2002 and has broadened with time to include multiple countries worldwide. The primary focus of SOAR is testing the two main CA-RTI pathogens: *S. pneumoniae* and *H. influenzae* and additionally *Streptococcus pyogenes* and *M. catarrhalis* in some centres. The pathogens are isolated from clinical specimens from outpatients with diagnosed CA-RTI and susceptibilities are tested in a central laboratory against a wide range of antibiotics. The results are analysed according to three recognized and standardized methodologies from the CLSI, EUCAST and using PK/PD criteria.^39^ The inclusion of three different breakpoints allows review of results according to the most appropriate breakpoint for local circumstances, as well as aiding comparison with other centres, countries or regions. The detailed results from SOAR and other international surveillance studies provide an evidence base for antimicrobial policies, inform antibiotic prescribing guidelines, and can be used as part of training concerning appropriate antibiotic prescribing. More recently, EUCAST has introduced dose-specific breakpoints which mean that it is now possible, using data derived from AST studies such as SOAR, to ascertain the effect of increasing the dose on the susceptibility of a specific pathogen.^40^

### Other activities

Since the publication of the Consensus Principles, awareness of the serious nature of the AMR threat has been growing. An early published concern at a high governmental level in the UK came in the 2011 Annual Report of the UK Chief Medical Officer (now UK Special Envoy on Antimicrobial Resistance) Professor Dame Sally Davies.^41^ The report reviewed AMR, setting out the response to the challenges and opportunities in the prevention, diagnosis and management of infectious diseases and included a series of recommendations framed as challenges for action. The report stressed the need for politicians in the UK to prioritize AMR as a major area of concern.

One of the key pillars in addressing rising AMR is knowledge of local susceptibility of the most-commonly occurring bacterial pathogens.^25^ Knowledge of the susceptibility of common pathogens can only be found through surveillance, which is, therefore, vital to inform clinical therapy decisions and, more broadly, to guide policy recommendations.^42–44^ In 2014, the WHO Antimicrobial Resistance Global Report on Surveillance^45^ provided, for the first time, an accurate picture of the magnitude of AMR and the current state of surveillance globally. One of the key findings was that there were significant gaps in surveillance, and a lack of standards for methodology, data sharing and coordination. In the same year, the UK Prime Minister commissioned a major AMR review that culminated in 2016 in the much-cited and publicized final report by Lord Jim O’Neill entitled ‘Tackling drug-resistant infections globally: Final report and recommendations’.^46^ The ten key recommendations from this report are shown in Table 1.

**Table 1.** Recommendations to address AMR

Tackling drug resistant infections globally, 2016^46^	WHO Global Action Plan on AMR, 2015^47^
Global public awareness campaign on AMR. Improve hygiene and sanitation to prevent the spread of infection. Reduce unnecessary use of antimicrobials in agriculture and their dissemination into the environment. Improve global surveillance of drug resistance in humans and animals. Promote new, rapid diagnostics to cut unnecessary use of antibiotics. Promote the development and use of vaccines and alternatives. Improve the numbers, pay and recognition of people working in infectious diseases. Establish a Global Innovation Fund for early-stage and non-commercial research. Better incentives to promote investment for new drugs and improving existing ones. Build a global coalition for real action.	Improve awareness of AMR. Strengthen knowledge through surveillance and research. Reduce the incidence of infection. Optimize use of antimicrobials. Sustainable investment in new medicines, diagnostic tools and vaccines.

In 2015, the WHO Worldwide Country Situation Analysis: response to antimicrobial resistance^48^ determined the extent that practices addressing AMR had been put in place, and where more work was needed. In the same year, the WHO produced the Global Action Plan (GAP) on AMR^47^ which laid out five clear objectives that were similar to the O’Neill report recommendations (Table 1). By 2018, 63 member countries had published national action plans (NAPs) for AMR containment and analysis of 52 of these revealed that 44 had considered establishing a national surveillance system.^49^ In a 2021 global survey of implementation of the GAP, over 90% of responding countries (*n *= 163) commented that COVID-19 had a negative effect on the development and implementation of NAPs to address AMR, with reasons including funding issues and the need to defer activities and campaigns due to the pandemic. Only around one-half of countries had a coordinated mechanism to prioritize, cost, implement and monitor NAPs but over 60% had linked their NAPs to other health topics or plans. In more than three-quarters of countries, in-service training relating to AMR was being provided to healthcare workers. Importantly, in terms of surveillance systems to monitor AMR, 72% of responding countries now had systems to collate data nationally for common bacterial infections in patients in both hospitals and the community.^50^

In 2017, the European Commission adopted the European Union (EU) One Health Action Plan against AMR.^51^ The plan comprised three main objectives: (1) making the EU a best-practice region; (2) boosting research, development and innovation; and (3) intensifying EU efforts worldwide to shape the global agenda on AMR and the related risks.^51^

The Davos Economic Forum Industry Declaration^52^ was signed in December 2017 by over 100 companies and associations actively engaged in combating AMR, pledging to work to reduce the development of AMR, to invest in research and development to meet public health needs with new innovative diagnostics and treatments and to improve access to high-quality antibiotics. These recommendations were coincident with those published after the high-level United Nations meeting on AMR in 2016.^53^ The AMR Benchmark Report, now in its second edition, evaluates pharmaceutical companies with a stake in the antibiotics market in areas where they could act to limit AMR, such as research and development, managing manufacturing waste and ensuring appropriate access and stewardship and provides a measure of how companies are improving the way they address AMR.^54^

Awareness of AMR is increasing globally and has been an important consideration during the ongoing COVID-19 pandemic. In January 2022, results from the Global Research on Antimicrobial Resistance (GRAM) study, as mentioned previously, were published in *The Lancet*.^33^ The global study estimated the disease burdens associated with, and attributable, to AMR and included data for 23 bacterial pathogens, and 88 pathogen–drug combinations.

### Antibiotic stewardship

The overall aim of the Consensus Principles when they were first compiled was to provide a clear and straightforward framework for physicians to achieve appropriate antibiotic prescribing.^25^ In order to assist in the development of tools for antibiotic stewardship at local, national and global levels which would, in turn, reduce AMR, in 2019 the WHO launched the Access, Watch, Reserve (AWaRe) classification of antibiotics.^55^ In the AWaRe classification, antibiotics are assigned to different groups emphasizing the importance of appropriate antibiotic prescribing and supporting antibiotic monitoring and stewardship activities.^55^ The ‘Access’ group includes antibiotics with activity against a range of common susceptible pathogens whilst also showing lower potential to cause resistance than those in the other groups. Examples include amoxicillin, amoxicillin/clavulanic acid, ampicillin and first-generation cephalosporins such as cefazolin and cefalexin. The ‘Watch’ group includes antibiotic classes that have higher resistance potential. Examples include macrolides such as azithromycin, clarithromycin, second- and third-generation cephalosporins such as cefuroxime, cefixime, ceftriaxone and fluoroquinolones such as ciprofloxacin. This group should be prioritized as key targets of stewardship programmes and monitoring. The ‘Reserve’ group includes antibiotics that must be reserved for treatment of infections due to multidrug-resistant (MDR) organisms and should be regarded as ‘last resort’ options. Examples include fosfomycin, colistin and linezolid. According to the WHO campaign ‘Adopt AWaRe. Handle antibiotics with care’ to promote responsible use of antibiotics, antibiotics from the ‘Access’ group should, by 2023, make up at least 60% of consumption, which would lead to improved use of antibiotics, lower costs and increased access.^55^

### Additional Principles

It has more recently been suggested that two further principles should be added to the original list, in order to reiterate some important issues. A seventh principle should stress that whilst prescribing may be empirical, it should be done intelligently,^56^ taking into account, in addition to guideline recommendations and local susceptibility information, if available, other factors such as any current safety warnings relating to any of the possible antibiotics or any other cautions that might have relevance to the prescribing options.

As an example, international guideline recommendations in CA-RTIs include macrolides and fluoroquinolones but in specific situations only. Macrolides are not recommended for empiric therapy given the high levels of resistance that are being observed; for example the IDSA Clinical Practice Guideline for Acute Bacterial Rhinosinusitis in Children and Adults states that macrolides (clarithromycin and azithromycin) are not recommended for empiric therapy for acute bacterial rhinosinusitis (ABRS) in children or adults due to high rates of resistance among *S. pneumoniae.*^57^ International guidelines for the treatment of CAP in adults recommend the use of macrolide antibiotics only in areas with pneumococcal resistance to macrolides of <25%.^58^ The recent widespread concern about the emergence of microbial azithromycin resistance due to unrestricted use during the COVID-19 pandemic^7^ has arisen from the fact that use of azithromycin results in rising levels of macrolide resistance^7,59^ and even before COVID-19, surveillance studies revealed reduced azithromycin susceptibility.^60^

Safety is another consideration when choosing the most appropriate antibiotic. This has become important when the use of fluoroquinolone antibiotics is under consideration. Fluoroquinolones are associated with disabling and potentially permanent side effects of the tendons, muscles, joints, nerves, and CNS that can occur together at the same time in the same patient.^61^ The FDA has issued safety warnings recommending limitation of the use of fluoroquinolones, particularly in ABRS, acute exacerbations of chronic bronchitis and uncomplicated urinary tract infection (UTI) when other treatments are available, because the risk of serious side effects generally outweighs the benefits in these patients.^61^ In addition, the CNS toxicity may be aggravated by concomitant non-steroidal anti-inflammatory use.^61–63^ A meta-analysis demonstrated that exposure to fluoroquinolones for the management of common upper or lower RTIs and also UTIs is substantially associated with aortic aneurysm and aortic dissection.^64^

It has also been considered useful to include an eighth, and final principle which should be to remind prescribing physicians to encourage their patients to comply with the prescription and to follow any instructions given to them.^56^ This is important to reiterate because lack of compliance can potentially lead to resistance as a result of exposure of the pathogen to sub-inhibitory concentrations of the antibiotic. In addition, this would lead to sub-optimal clinical outcomes due to a lower than optimal dose.^56^

## Conclusions

The review of multiple and ongoing global initiatives and the continued rise in AMR in the 20 years since their original publication, show that adherence to the original Consensus Principles for appropriate antibiotic prescribing, along with the additional two principles, is as important now as it was in 2002. Achieving appropriate antibiotic prescribing in the management of patients within the community, in particular those suffering from CA-RTIs, is a vital objective in order to ensure that the right patient receives the right antibiotics at the right time to achieve optimal clinical outcomes while at the same time helping to limit further increases in AMR.

Against this background of developments since they were first outlined in 2022, the Consensus Principles are still needed today to ensure the appropriate use of antibiotics. The inclusion of two additional principles has broadened their scope to cover other factors such as safety and also to make sure patient compliance is addressed. The updated Consensus Principles can be summarized as:

1. Treat only bacterial infections.
2. Optimize diagnosis and severity assessment.
3. Maximize bacterial eradication.
4. Recognize (and act on) local resistance prevalence.
5. Utilize pharmacodynamics to assist in the choice of effective agents and dosing.
6. Integrate local resistance, efficacy and cost-effectiveness.
7. Prescribe empirically, but intelligently.
8. Encourage patient compliance.

### Objective of this Supplement: investigation of the AMR response at a national level

As outlined in this paper, there has been an extensive and ongoing global response to rising AMR which the development of the Consensus Principles aims to address. Whilst GAPs for AMR are in place, along with the availability of a range of comprehensive international guidelines and recommendations, it is also helpful to have a clear idea of how these are being actioned in different countries around the world; for example, has a NAP for AMR been instigated and put into practice or are there local AST studies in place. In this Supplement, in order to obtain a wide-ranging picture, a series of situation analyses and investigations have been carried out for nine disparate countries, namely Brazil, India, Kuwait, Mexico, Pakistan, Russia, Saudi Arabia, Türkiye and Vietnam, and an individual paper prepared summarizing the findings for each one. These analyses have covered, where available, information on antibiotic use and prescribing, approach to AMR, availability of local susceptibility data, use of international and/or local management guidelines and how these link to antibiotic availability. In addition, it is also pertinent to understand how the situation impacts patient management and the functions of the clinical microbiology laboratory and so, where possible, the views of clinicians and clinical microbiologists have been included along with identification of areas where more information is required. Summaries of the key points from these analyses covering the areas mentioned above are the basis of the nine accompanying papers in this Supplement, which provides a call to action to improve both the clinical outcome for patients and to minimize further rises in AMR.
